# BRCA1‐associated‐protein‐1 inactivated melanocytic tumours: characterisation of the clinicopathological spectrum and immunohistochemical expression pattern of preferentially expressed antigen in melanoma

**DOI:** 10.1111/his.15318

**Published:** 2024-09-13

**Authors:** Yitong Xu, Alejandro A Gru, Thomas Brenn, Katharina Wiedemeyer

**Affiliations:** ^1^ Department of Pathology and Laboratory Medicine University of Calgary Calgary AB Canada; ^2^ Department of Pathology University of Virginia Charlottesville VA USA; ^3^ Department of Pathology New York–Presbyterian Hospital/Columbia University Irving Medical Center New York USA; ^4^ Arnie Charbonneau Cancer Institute, Cumming School of Medicine, University of Calgary Calgary AB Canada; ^5^ Department of Pathology University of Michigan Ann Arbor MI USA

**Keywords:** atypical Spitz, BAP1, germline mutation, melanocytic tumour, melanoma, PRAME

## Abstract

**Aims:**

BRCA1‐associaed protein‐1 (BAP1) inactivated tumours (BIMT) are rare melanocytic tumours that may be mistaken for Spitz tumours or melanoma. They occur sporadically or in association with the BAP1 tumour predisposition syndrome (BAP1–TPDS), which may be complicated by uveal or cutaneous melanoma, mesothelioma, basal cell carcinoma and renal cell carcinoma. The aim of this study was to characterise the clinicopathological features and the immunohistochemical expression pattern of preferentially expressed antigen in melanoma (PRAME) of BIMT in a large patient cohort.

**Methods and results:**

Ethical approval was obtained, haematoxylin and eosin‐stained slides were reviewed, PRAME immunohistochemistry was performed and clinical follow‐up was obtained from patient records. Sixty‐five BIMT from 38 patients (F:M = 4.4:1) were identified. BIMT were typically located on the trunk and head and neck (median size = 0.5 cm). Seven patients with BAP1–TPDS (range = 16–66 years, median = 25) had multiple BIMT (median = 5), while sporadic BIMT were solitary (median patient age = 39 years). One of seven patients with BAP1–TPDS developed additional malignancies (mesothelioma and cutaneous spindle cell melanoma) and died of complications of mesothelioma. All other patients are alive without recurrence of BIMT (median follow‐up = 42 months). BIMT presented as intradermal, nodular aggregates of epithelioid melanocytes with low mitotic activity and moderate to severe cytological atypia in 63% of cases. A background conventional naevus was present in 64%. PRAME immunohistochemistry showed negative or weakly patchy positive staining in all BIMT.

**Conclusions:**

BIMT are more common in a sporadic setting and behave indolently, despite worrying cytological atypia. PRAME immunohistochemistry is a reassuring tool in distinguishing BIMT from melanoma.

## Graphical abstract

BAP1‐inactivated tumours (BIMT) are indolent proliferations of dermal epithelioid melanocytes with nuclear loss of BAP1, and can present with severe cytological atypia. PRAME (preferentially expressed antigen in melanoma) immunohistochemistry consistently shows patchy and weak staining in BIMT and serves as a reassuring tool to distinguish BIMT from melanoma.

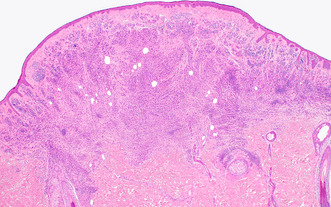

**Abbreviations**
BAP1: BRCA1‐associated protein
BAP1‐TPDS: BAP1 tumour predisposition syndrome
BIMT: BAP1 inactivated tumours
BRCA: breast cancer gene
PRAME: preferentially expressed antigen in melanoma

## Introduction

BAP1‐inactivated melanocytic tumours (BIMT), also referred to as BAP1‐inactivated melanocytomas, present clinically as skin‐coloured, dome‐shaped papules typically effecting trunk, head and neck area and extremities.^1^ Histologically, they are dermally based and show an epithelioid cell morphology with varying degrees of cytological atypia.^2^ BAP1 stands for BRCA1‐associated protein 1 and is encoded on chromosome 3 (locus 3q21.1). It functions as a tumour suppressor gene that is implicated in DNA damage response, transcriptional regulation and chromatin modulation.^3^ Germline mutations in BAP1 result in an autosomal dominant tumour predisposition syndrome that is associated with a high risk of developing various tumours, including BIMT, cutaneous melanoma, uveal melanoma, mesothelioma, renal cell carcinoma, lung adenocarcinoma and meningioma.^2^ BIMT are often the first symptom of the tumour predisposition syndrome, and histopathological recognition is therefore important to guide patient management.^4^ The presence of varying degrees of cytological atypia in BIMT poses a diagnostic challenge to distinguish the entity from atypical Spitz tumours and melanoma.^4^, ^5^ PRAME (preferentially expressed antigen in melanoma) is a cancer testis antigen that has demonstrated valuable utility in assisting in differentiating melanoma from benign counterparts, given its high specificity of diffuse expression in melanoma and absence to low expression in benign melanocytic naevi.^6^, ^7^ The PRAME expression profile has not been comprehensively investigated in BIMT. The aims of this study were to (1) describe the clinical and histopathological spectrum of BIMT in a large patient cohort in Southern Alberta, Canada, (2) study the behaviour of BIMT by providing long‐term follow‐up and (3) study the expression pattern of PRAME in BIMT.

## Materials and methods

Ethical approval was obtained from the health research ethics board of Alberta (HREBA.CC‐19‐0379). Haematoxylin and eosin‐stained sections of 65 BAP1‐inactivated melanocytic tumours were retrieved from the departmental files of the Alberta Precision Laboratories, Calgary, Alberta, Canada. BAP1 inactivation was defined as complete loss of nuclear staining of the immunohistochemical marker BAP1.^8^ The histological features were reviewed and the following histopathological criteria were documented: tumour circumscription, junctional and dermal component, pigmentation, stromal fibrosis, cytological atypia (mild, moderate, severe), mitotic activity, necrosis, infiltrative growth, inflammatory infiltrate and existence of a conventional melanocytic in the background. Clinical data, including genetic test results for BAP1 germline mutations and follow‐up, were obtained from patient records. All patient records were reviewed for additional cancer diagnoses and additional skin excisions. Immunohistochemistry with adequate controls was performed for S‐100, Sox10, MelanA, HMB45, p16, BAP1 and Ki‐67, according to the manufacturer's instructions (Table 1). In a subset of cases, immunohistochemical staining had been performed for routine work‐up, and in these cases the slides were reviewed without repeating the stains. Immunohistochemistry for PRAME was performed on 4‐μm‐thick formalin‐fixed paraffin‐embedded whole tissue sections following pressure cooker antigen retrieval (Target Retrieval Solution; pH 6.1 citrate buffer; Dako, Carpinteria, CA, USA) using a rabbit anti‐PRAME monoclonal antibody (1:100 dilution; clone EPR20330; Biocare Medical, Pacheco, CA, USA); the Novolink polymer detection system (Leica, Buffalo Grove, IL, USA) was used. Nuclear staining was assessed and given as the percentage of overall tumour cells (0%: 0; 1–25%: 1, 26–50%: 2; 51–75%: 3, 76–100%: 4) and staining intensity (ranging from 0 to 4 as follows: absent: 0; weak: 1; moderate: 2; strong: 3). A combined score was calculated as the sum of the quantity and staining intensity scores.^9^ The normal peritumoural tissue served as positive control.^10^ The source of the antibodies and their dilutions are listed in Table 1.

**Table 1:** Lists all antibodies used with their source and dilutions

Antibody	Clone	Dilution	Source
S100	POLY	RTU	Dako North America, Carpinteria, CA, USA
BAP1	C4	1:150	Santa Cruz Biotechnology, Santa Cruz, CA, USA
SOX10	BC34	1:100	Biocare, Pacheco, CA, USA
HMB45	HMB45	1:100	Dako North America, Carpinteria, CA, USA
MelanA	A103	1:75	Dako North America, Carpinteria, CA, USA
Ki‐67	OTI3B6	1:150	Origene, Rockville, MD, USA
P16	E6H4	1:24	CINtech, Seattle, WA, USA
PRAME	EPR20330	1:100	Biocare Medical, Pacheco, CA, USA

## Results

### Clinical features

Our histopathological archives for BAP1‐inactivated tumours were searched from 2010 to 2022. The Calgary health zone includes an average of 1 500 000 people. Sixty‐five BIMT involving 31 female and seven male patients (ratio f:m = 4.4:1) were included in the study. With 38 patients affected by BIMT the estimated prevalence of BIMT in the Calgary health zone is 0.000025. With seven patients carrying a BAP1 germline mutation the prevalence of BAP1 germline mutations presenting with BIMT is approximately 0.0000047 in this cohort. The patient age ranged from 16 to 77 years with a mean of 39.6 years; two patients were younger than 18 years. All tumours were completely excised by primary excision or re‐excision. Seven patients (18.4%) had a BAP1 germline mutation. These patients presented at a younger age (range = 16–66, median = 25 years) without sex predilection (four females, three males). The results of the genetic analyses with documentation of the specific mutations were available for six patients and included the following: c.376‐2A>G, c.376‐2_392del, c.1717delCp.(Leu573TrpfsTer3), c.458_549delCT, c.1358_1359del and c.485_495delCT. The majority of BIMT were located on the trunk (*n* = 26, 43%, including 15 BIMT located on the back, three on the shoulder, four on the chest and four on the abdomen) and on the head and neck area (*n* = 26, 43%, 11 BIMTs on the face, seven on the ears, five on the neck and three located on the scalp). The remaining tumours were located on the extremities (*n* = 13, seven located on the upper extremities, six on the lower extremities including acral sites). The tumours presented with a median size of 0.55 cm (range = 0.2–1.5 cm, available for *n* = 22). Patients with BAP1 germline mutations presented frequently with multiple BIMT (range of number of BIMT per patient 1–8, mean 6). The anatomical distribution did not differ significantly between germline‐associated and sporadic tumours, nor did the size. No recurrences or metastases of BIMT were noted in the entire cohort (follow‐up period = 4–111 months, mean = 44 months). One male patient with BAP1 germline mutation died of complications of mesothelioma at the age of 69 years, 42 months after the diagnosis of one BIMT. This patient's history is also remarkable for two basal cell carcinomas and an invasive melanoma that the patient developed 3 years prior to his mesothelioma. The melanoma (size 1.5 × 1.4 cm) was amelanotic, showed spindle cell morphology and a maximum tumour thickness of 2.1 cm. None of the remaining patients with BAP1 germline mutations developed a malignant tumour diagnosis during the follow‐up period (follow‐up period range = 8–111 months, mean = 49 months). According to the medical records, 26 patients also had a history of conventional melanocytic naevi unrelated to their BIMT(s). The number of conventional melanocytic naevi ranged from one to 28 per patient. Within the group of patients with sporadic BIMT, the mean number of additional conventional melanocytic naevi was four per patient. Within the group of patients with BAP1 germline mutations, the mean number of additional conventional naevi was 5.5. Within the group of patients with sporadic BIMT (patient *n* = 31), one patient had a remote history of melanoma (no histopathological data available), two had a history of basal cell carcinoma, one patient had diffuse large B cell lymphoma, one patient revealed a history of Hodgkin lymphoma plus a history of ductal carcinoma *in‐situ* of the breast, one patient developed invasive ductal carcinoma of the breast and one patient had a remote history of prostatic adenocarcinoma plus a remote history of melanoma. Two patients with sporadic BIMT harboured BRCA germline mutations (one BRCA1 and one BRCA2 mutation). The clinical characteristics of all BIMT are summarised in Table 2.

**Table 2:** Summary of the clinical data on sporadic and germline‐associated BIMT

BAP1‐deficient tumours (BIMT)	Number of patients	Number of BMIT	Patient age (years)	Sex	Size (cm) *n* = 36	Localisation	Follow‐up (months) mean	Outcome
Sporadic	31	32	41 (median)	F = 27 M = 4	*n* = 15 Range: 0.15–1.1 0.5 (median)	Head & neck: *n* = 16 Trunk: *n* = 9 Extremities: *n* = 7	40.7	No recurrences/metastases/ deaths
Associated with BAP1‐germline mutation	7	33	25 (median)	F = 4 M = 3	*n* = 21 Range: 0.2–1.5 0.5 (median)	Trunk: *n* = 16 Head & neck: *n* = 11 Extremities: n = 6	49	No recurrences/metastases 1 death*
Total	38	65	Range: 16–77 39 (median)	F = 31 M = 7 Ratio F:M: 4.4: 1	Range: 0.15–1.5 0.5 (median)	Trunk: *n* = 25 Head and neck: *n* = 27 Extremities: *n* = 13	Range: 4–111 44 months (mean) 43 months (median)	No recurrences/metastases 1 death*

* Death due to complications of advanced mesothelioma. BIMT, BAP1 inactivated melanocytic tumours.

### Histological features

**Figure 1:**
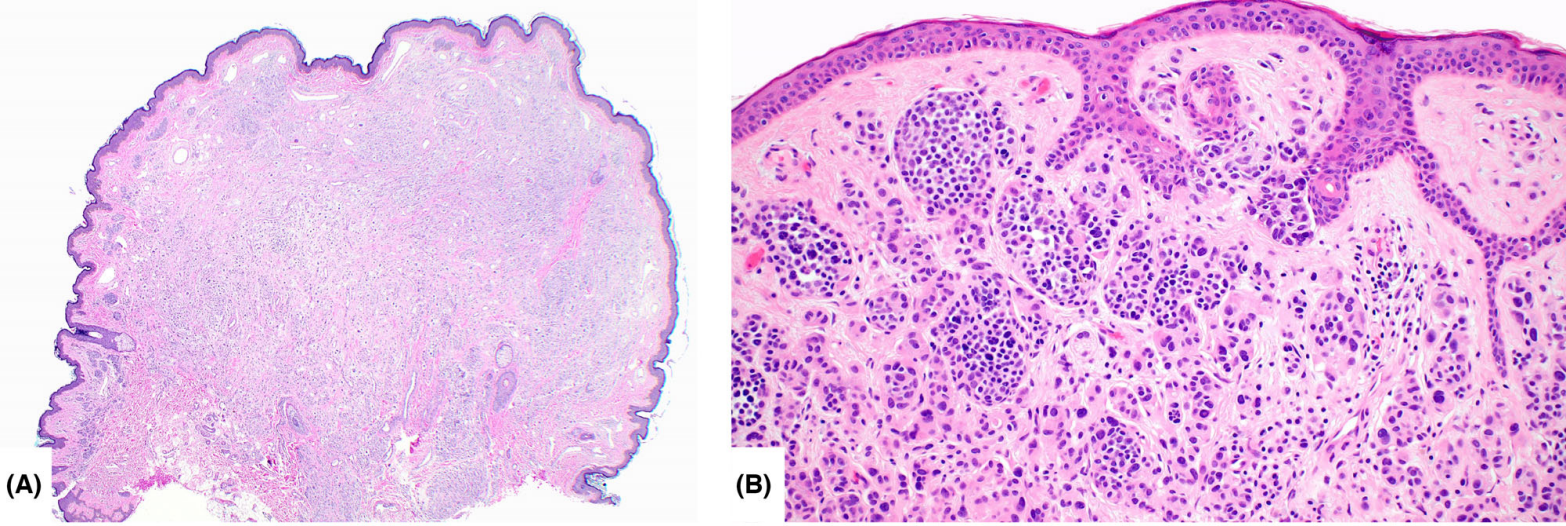
**A,** BIMT presenting as a dome‐shaped, well‐circumscribed dermal proliferation of large epithelioid cells arranged in nests and sheets. Flanking the main tumour on the right are bland nests composed of smaller naevus cells representing a conventional background naevus. **B,** A subtle junctional component comprised of small single and small nests of epithelioid melanocytes is present. Junctional components in BIMT were found in 17 tumours (26%) to a varying extent, but most commonly minor. [Color figure can be viewed at wileyonlinelibrary.com]

**Figure 2:**
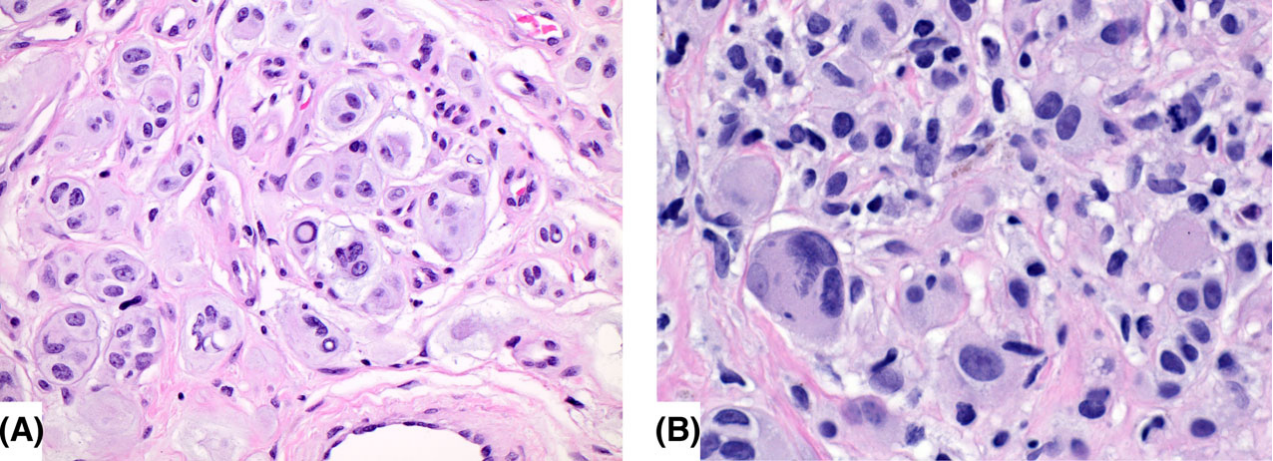
**A,** Dermal nests of large epithelioid melanocytes with moderate cytological atypia: The nuclei show pleomorphism, finely dispersed chromatin with prominent nucleoli. Nuclear pseudo‐inclusions are a common finding. A single mitotic figure is appreciated. **B,** Multinucleation and severe cytological atypia, including a higher degree of nuclear pleomorphism and hyperchromasia are seen No necrosis is present. The mitotic activity remains low. [Color figure can be viewed at wileyonlinelibrary.com]

**Figure 3:**
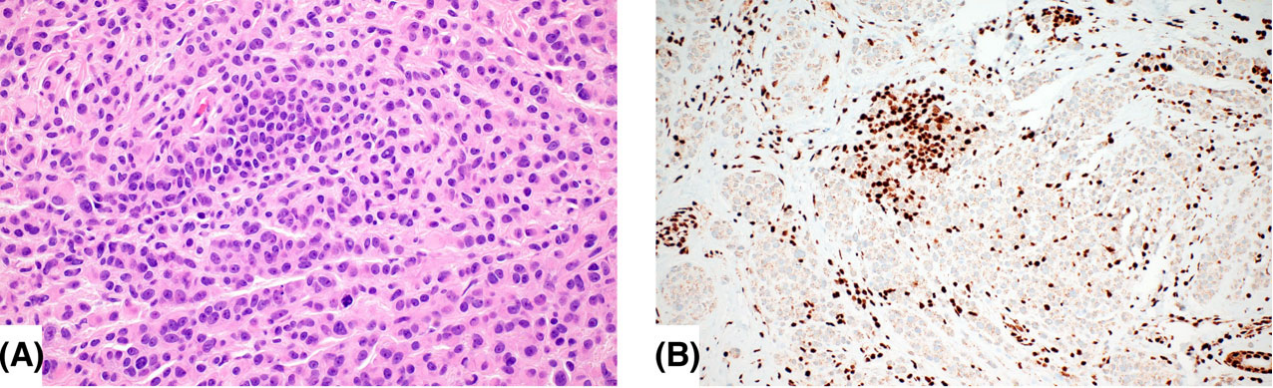
**A,** Example of a BAP1 inactivated melanocytic tumour (BIMT) intermingling with the conventional background naevus. Most BIMT show a sharp demarcation of the banal naevus that is usually flanking the BIMT (see Figure 1a). The cytological features of BIMT are subtle (epithelioid morphology and prominent nucleoli). Note the size difference of the two cell populations. In **B**, BAP1 immunohistochemistry highlights the conventional naevus cells that show nuclear positivity. The majority of cells show loss of nuclear BAP1. [Color figure can be viewed at wileyonlinelibrary.com]

All BIMT were well‐circumscribed, nodular tumours located within the superficial and mid‐dermis (Figure 1A). Twenty‐six tumours (40%) showed a minor junctional component consisting of small melanocytic nests composed of epithelioid melanocytes and few single epithelioid cells (Figure 1B). The tumours were composed of nests and sheets of non‐pigmented (37 tumours, 57%) or lightly pigmented (28 tumours, 43%) epithelioid cells with amphophilic cytoplasm and round to ovoid nuclei with evenly dispersed chromatin and prominent nucleoli (Figure 2A). Moderate to severe cytological atypia, including irregular nuclear contours, nuclear pseudoinclusions, bizarre‐formed nuclei, multinucleation and hyperchromasia, was present in 41 tumours (63%) (Figure 2B). No dermal mitoses were observed in the majority of BIMT (80.2%). Low mitotic activity was observed in seven tumours (10.8%), ranging from one to two mitoses per mm^2^, but no atypical mitotic figures were identified. No foci of tumour necrosis were observed in any tumour. A brisk lymphocytic inflammatory infiltrate was present in seven tumours (10.8%), a mild to moderate inflammatory infiltrate in 35 cases (53.8%) and no inflammation was seen in 23 BIMT (35.4%). Only one germline‐associated tumour showed significant stromal fibrosis; the remainder of cases had no remarkable fibrosis. One germline‐associated BIMT showed angiomatoid features with multiple small, dilated vessels intermingling with the epithelioid melanocytes. A conventional background naevus flanking the BAP1 inactivated proliferation on one or both sides was present in majority of the tumours (*n* = 50, 76.9%). The background naevus made up the lesser part of the tumours in all cases; the dominant component was the dermal BAP1‐inactivated proliferation. The background conventional naevus was composed of dermal melanocytic nests only (33 cases), but also revealed compound architecture in 17 cases. Two cases showed intermingling of the banal, smaller naevus cells with the large, epithelioid BAP1‐inactivated tumour cells (Figure 3A,B), whereas most tumours had a clear demarcation of conventional naevus and BAP1‐inactivated cells. The diagnosis of the cutaneous invasive melanoma arising in one patient with BAP1 germline mutation was straightforward histopathologically. The tumour was composed of atypical spindle cells arranged in fascicles and sheets within dermis demonstrating an infiltrative growth pattern and a high mitotic rate (20 per mm^2^). No adjacent conventional naevus nor a BIMT was present in the periphery of the melanoma.

### Immunohistochemistry

**Figure 4:**
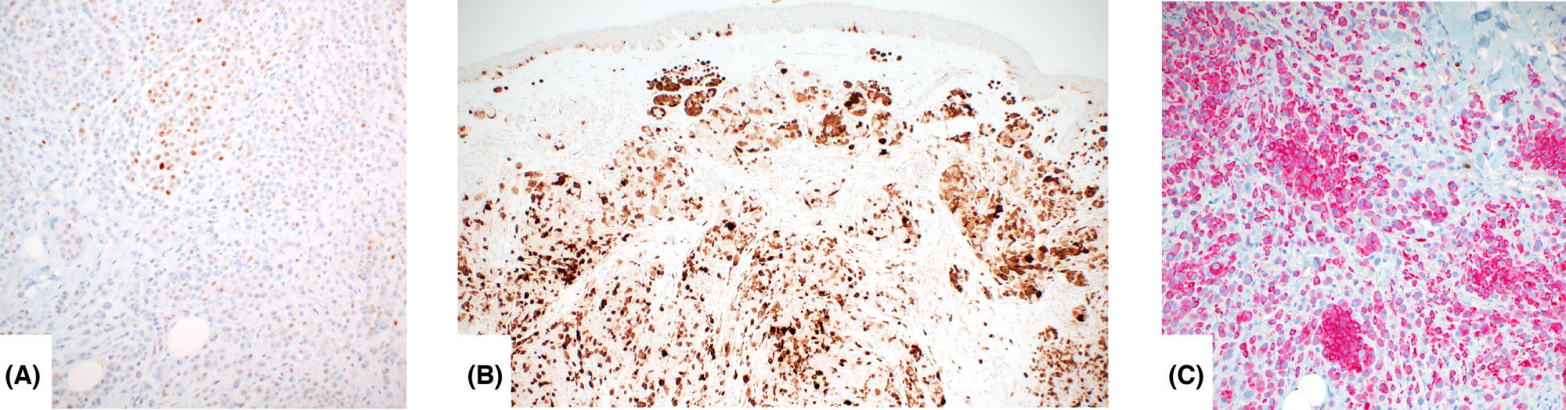
Preferentially expressed antigen in melanoma (PRAME) immunohistochemistry can show nuclear weak, patchy positivity in BIMT (**A**). P16 is retained (**B**) or demonstrates mosaic staining pattern in BIMT. MelanA shows strong and diffuse cytoplasmic staining (red) but the proliferative activity is low (Ki‐67 (brown) stains rare single cells, < 1%) (**C**). [Color figure can be viewed at wileyonlinelibrary.com]

All tumours were strongly and diffusely positive for S100 (nuclear and cytoplasmic), Sox10 (nuclear), MelanA (cytoplasmic) (Figure 4C) and negative for HMB45, except for a junctional component in the conventional background naevi. Nuclear p16 staining (available for 37 cases) was either retained (19 cases, 51.4%) or mosaic^16^ (Figure 4B). PRAME showed focal or patchy, weak nuclear staining in all tumours (Figure 4A). The overall combined score was low with a mean of 3 (range = 0–80), quantity range = 0–40% of tumour cells, intensity range = 0–2). Ki‐67 staining revealed a low mitotic index (Figure 4C) in all BAP inactivated tumours. The melanoma arising in the patient with BAP1 germline mutation showed loss of nuclear BAP1 staining but diffusely positive cytoplasmic staining, and PRAME showed focal nuclear expression.

## Discussion

Prior to their first description in 2011 by Wiesner *et al*. BIMT had been classified as epithelioid Spitz tumours or melanomas.^11^ Since 2011, more information and details concerning the histopathological features, the pathogenesis and the genetic background of BIMT have been gathered.^12^ BIMT show a bi‐allelic inactivation of the BAP1 tumour suppressor gene located on chromosome 3q21, which can be caused by loss‐of‐function mutations or by deletion affecting the BAP1 locus. BIMT typically arises from a conventional naevus with BRAFp.V600E or NRAS mutations or RAF1 fusion.^1^, ^3^, ^13^ The double hit results in a clonal expansion of the BAP1‐inactivated clone with the typical epithelioid phenotype. In the sporadic setting, the double hit is caused by loss‐of‐function mutations altering the BAP1 nucleotide sequence often combined with a chromosomal deletion involving the wild‐type BAP1 locus. In patients with BAP1 germline mutations, the second hit is the inactivation of the remaining wild‐type BAP1 allele.^1^, ^3^, ^13^ Despite this pathogenetic insight, data regarding the prevalence and the behaviour of BIMT have been scarce. Approximately 200 families with BAP1 germline variants have been described to date.^14^ In our cohort, seven patients presenting with BIMT carried a BAP1 germline mutation, and we calculated the prevalence to be approximately 0.0000047 in the Calgary health zone. The overall prevalence of BIMT in out cohort was 0.000025, stressing that BIMT occur more commonly in the sporadic setting than in the syndromic setting. Our data confirm that BIMT are often the primary manifestation in patients carrying a BAP1 germline mutation, and that these patients present with multiple BIMT at a young age, commonly within the second decade of life, as reported previously.^12^ The diagnosis of a single BIMT in a patient does not imply genetic testing for a BAP1‐germline mutation unless multiple BIMT are seen in the same patient or there is clinical suspicion due to a positive family history or manifestation of other tumours, especially uveal melanoma, cutaneous melanoma, mesothelioma and renal cell carcinoma.^14^ Malignant transformation of BIMT has been reported in both sporadic and germline‐associated tumours,^15^, ^16^ but the majority of BIMT show an indolent behaviour.^11^ Our data stress the indolent behaviour of BIMT in both settings, sporadic and syndromic. None of the tumours showed recurrence or aggressive behaviour despite worrisome histopathological features. No malignant transformation was noted in any of the BIMT in this study, which argues against BIMT being a significant melanoma precursor lesion. The spindle cell melanoma arising in one of the patients with BAP1 germline mutation did not show any histopathological resemblance with BIMT, despite loss of nuclear BAP1 by immunohistochemistry, and no conventional naevus or a BIMT was present in the background. The cytoplasmic expression of BAP1 in this melanoma is a finding of unknown significance. Cytoplasmic expression of BAP1 has been described in a subset of uveal melanomas, suggesting a functional role of BAP1 within the cytoplasm that warrants further investigation.^8^

Histopathologically, the classic morphology of BIMT is described as a biphasic growth pattern with a nodular or sheet‐like proliferation of epithelioid melanocytes in the background of an adjacent conventional naevus component.^17^ The larger epithelioid cells typically display abundant amphophilic cytoplasm with vesicular nuclei and prominent nucleoli, resembling the melanocytes seen in Spitz naevi.^3^ Other rare morphological features including rhabdoid, adipocyte metaplasia and nuclear pseudo‐inclusions have also been described.^15^, ^18^, ^19^ In a larger histopathological study conducted by Garfield *et al*.,^12^ a significant association of an extensive junctional component in BIMTs arising in patients with germline BAP1 mutations was observed. In our study, we did not see any significant histopathological differences between BIMT arising in the sporadic versus germline‐associated setting; in particular, a more prominent junctional component was occasionally present in both groups. According to our observations, germline‐associated BIMTs can present purely dermally or with a junctional involvement in the same patient. Pagetoid spread was observed in one single BIMT, occurring in a 16‐year‐old female with BAP1 germline mutation on the upper back. A prominent lymphohistiocytic infiltrate has frequently been described as a typical feature of BIMT.^1^ In our study we only saw a brisk lymphohistiocytic infiltrate in 10.8% of tumours, a mild lymphohistiocytic infiltrate in 40% of cases and no inflammatory infiltrate in 40.2%. The review of all patient records in our cohort reveals that patients with BIMT also commonly develop multiple banal conventional naevi without significant atypia, and only one patient had a history of primary cutaneous melanoma. Two patients with sporadic BIMT also had breast carcinomas and harboured BRCA mutations, which is probably a coincidental finding. The same assumption applies to the other malignant diagnoses in patients with sporadic BIMT in this cohort.

PRAME immunohistochemistry achieved low combined scores of quantity and intensity in all BIMT in this study. Previous data on PRAME expression in BIMT are limited. Lopez *et al*. studied five BIMT, none of which had an immunoreactivity score greater than 1+ (staining of 1 to 25% of tumour cells), and all cases demonstrated a weak staining intensity.^20^ In another study, Turner *et al*. reviewed PRAME immunohistochemistry in a small number of BIMT. In their study, diffuse PRAME positivity (defined as at least weak nuclear positivity in > 75% of atypical cells) was present in two of five cases. The remaining three cases showed non‐diffuse or negative PRAME staining.^21^ Both studies used a different PRAME antibody and incubation protocol compared to our study. Taken together, the data suggest that diffuse PRAME positivity in BIMT is a rare phenomenon.

## Conclusion

BIMT are indolent tumours characterised by large dermal epithelioid melanocytes with nuclear loss of BAP1, and present with a variable amount of cytological atypia and low mitotic activity. A conventional background naevus is present in > 75% of cases that should not mislead to a diagnosis of melanoma arising in a naevus. Malignant transformation of BIMT was not noted. Most BIMT occur sporadically as single tumours in patients who may also develop conventional melanocytic naevi without significant atypia. When arising in patients with BAP1 germline mutation, BIMT are often multiple and affect patients in their second decade of life. Cutaneous melanoma arising in patients with BAP1 germline mutation develop *de‐novo* without precursor in majority of patients. PRAME consistently shows patchy and weak staining in BIMT and serves as a reassuring tool to distinguish BIMT from melanoma.

## Author contributions

Yitong Xu: data collection, analysis and interpretation of results. Alejandro A Gru: immunohistochemical staining and interpretation of results. Thomas Brenn: study conception and design, analysis and interpretation of results. Katharina Wiedemeyer: study conception and design, interpretation of results and manuscript preparation.

## Conflicts of interest

The authors have nothing to disclose.

## Data Availability

The data that support the findings of this study are available from the corresponding author upon reasonable request.
